# Self-Healable and Recyclable Biomass-Derived Polyurethane Networks through Carbon Dioxide Immobilization

**DOI:** 10.3390/polym13244381

**Published:** 2021-12-14

**Authors:** Seohyun Baek, Juhyen Lee, Hyunwoo Kim, Inhwan Cha, Changsik Song

**Affiliations:** Department of Chemistry, Sungkyunkwan University, Suwon 16419, Korea; backgo04@gmail.com (S.B.); wngusqq@naver.com (J.L.); khw2651@naver.com (H.K.); ddrfgt@hotmail.com (I.C.)

**Keywords:** biomass, carbon dioxide, covalent adaptive network

## Abstract

Due to growing environmental issues, research on carbon dioxide (CO_2_) use is widely conducted and efforts are being made to produce useful materials from biomass-derived resources. However, polymer materials developed by a combined strategy (i.e., both CO_2_-immobilized and biomass-derived) are rare. In this study, we synthesized biomass-derived poly(carbonate-co-urethane) (PCU) networks using CO_2_-immobilized furan carbonate diols (FCDs) via an ecofriendly method. The synthesis of FCDs was performed by directly introducing CO_2_ into a biomass-derived 2,5-bis(hydroxymethyl)furan. Using mechanochemical synthesis (ball-milling), the PCU networks were effortlessly prepared from FCDs, erythritol, and diisocyanate, which were then hot-pressed into films. The thermal and thermomechanical properties of the PCU networks were thoroughly characterized by thermogravimetric analysis, differential scanning calorimetry, dynamic (thermal) mechanical analysis, and using a rheometer. The self-healing and recyclable properties of the PCU films were successfully demonstrated using dynamic covalent bonds. Interestingly, transcarbamoylation (urethane exchange) occurred preferentially as opposed to transcarbonation (carbonate exchange). We believe our approach presents an efficient means for producing sustainable polyurethane copolymers using biomass-derived and CO_2_-immobilized diols.

## 1. Introduction

Due to global warming and environmental pollution, research on the use of carbon dioxide (CO_2_) is broadly conducted alongside efforts to reduce carbon emissions via CO_2_ capture and storage [1,2,3,4,5]. This relatively inert gas can be efficiently converted into polymerizable platforms (monomers) and polymers, as well as many commodity chemicals such as methanol, carboxylic acid, and dimethyl carbonate [6,7,8]. For example, 5 or 6-membered cyclic carbonates, which can undergo ring-opening polymerizations, have been prepared by ring-closing reactions of 1,2 or 1,3-diols (or their equivalents) with CO_2_ [9,10,11,12], or by the cycloaddition of CO_2_ to ethylene oxide and other oxiranes [13,14,15,16]. Direct copolymerization methods (i.e., CO_2_ as a comonomer) have also been developed, e.g., ring-opening copolymerization with oxirane monomer(s) [17,18,19], and step-growth polymerizations with diols or dihalides [2,20]. However, synthesizing polymer or polymer platforms from biomass-based materials by directly introducing CO_2_ is rare. Although some biomass-derived oxiranes or aliphatic alcohols (diols) will be compatible with the conversion reaction or direct copolymerization of CO_2_ [21,22], furan-based biomass-derived monomers, such as 5-(hydroxymethyl)furfural (HMF) and 2,5-bis(hydroxymethyl)furan (BHMF) have not been utilized in CO_2_ immobilization.

**Figure 1:**
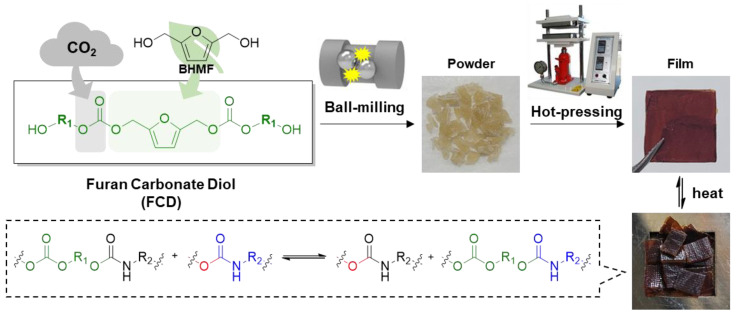
Synthesis of ecofriendly CO_2_-immobilized poly(carbonate-co-urethane) (PCU) networks and the preparation of the PCU films using a hot press.

Biomass feedstocks are ecofriendly and sustainable alternative resources and include forestry and crop residues, algae, municipal, and other organic wastes that can be used directly as fuel or converted into other reusable forms [23,24,25,26]. For example, cellulosic biomass can be converted into alcohols and acids, such as HMF, 2,5-furandicarboxylic acid, and isosorbide, from which various ecofriendly polymers can be synthesized [27,28,29]. Other lignocellulosic sugar alcohols, such as sorbitol, mannitol, xylitol, and erythritol, are also potential fuels and chemicals that are widely used in ecofriendly polymers and pharmaceutical applications [30,31,32,33,34,35,36]. We aimed to develop furan carbonate diols (FCDs) by directly immobilizing CO_2_ into furan-based, biomass-derived diols such as BHMF (Figure 1). These FCDs could then be further utilized to produce urethane-based network polymers, i.e., poly(carbonate-co-urethane) (PCU) networks. To the best of our knowledge, there are no prior reports on the production of network polyurethane copolymers using biomass feedstock and CO_2_ simultaneously.

Network polyurethanes (NPUs) are generally synthesized from a polyol as a network structure and have many applications because of their good elastic properties, thermal stability, and solvent resistance [37,38,39,40]. However, conventional NPUs are thermosets and cannot be recycled. Interestingly, these NPUs (and other network polymers) can acquire malleability and recyclability using covalent-bond (i.e., urethane) exchange reactions. Such network polymers are broadly defined as covalent adaptable networks (CANs) and enabled by dynamic covalent chemistry (DCC) such as transesterification, transcarbonation (carbonate exchange), transcarbamoylation (urethane exchange), and silyl ether exchange [41,42,43,44]. Generally, CANs are known to operate through two types of mechanisms, i.e., dissociative and associative processes [45,46]. Dissociative CANs can be depolymerized in thermal equilibrium states and then repolymerized, which may result in the loss of their crosslinking density and mechanical modulus. Conversely, associative CANs continuously maintain their crosslinking density under thermal equilibrium during a dynamic bond exchange. Research on the development of CANs using biomass-based monomers is actively being conducted [47,48], providing reprocessable, recyclable, and self-healable polymer networks through DCC [48,49,50]. However, cases of CANs using a biomass-based, CO_2_-immobilized platform are rare.

In this study, we prepared biomass-derived urethane-based network polymers using a FCD, a new platform derived from the direct immobilization of CO_2_ into BHMF [2,51,52,53]. The synthesized PCU network polymers were fabricated into films (Figure 1) and their thermal and thermomechanical properties were thoroughly characterized. Using a model compound study, we demonstrated that the thermally-induced reprocessing and self-healing properties of the PCU films arose due to the dynamic covalent exchange of urethane bonds, rather than carbonates. We believe our strategy using the simultaneous use of biomass and CO_2_ will be useful for the development of sustainable polymer materials.

## 2. Materials and Methods

### 2.1. Materials

All chemicals used in the experiments were purchased from commercial sources: 2,5-bis(hydroxymethyl)furan (Nanjing Sunshine Chemical Co., Ltd., Nanjing, China), 6-Bromo-1-hexanol (TCI, Tokyo, Japan), 3-Bromo-1-propanol (Sigma Aldrich, St. Louis, MO, USA), 1,4-Benzenedimethanol (TCI, Tokyo, Japan), tert-Butyl(chloro)diphenylsilane (Sigma Aldrich, St. Louis, MO, USA), imidazole (Alfa Aesar, Ward Hill, MA, USA), erythritol (TCI, Tokyo, Japan), hexamethylene diisocyanate (TCI, Tokyo, Japan), 4,4′-diisocyanato-methylenedicyclohexane (TCI, Tokyo, Japan), cesium carbonate (TCI, Tokyo, Japan), tetrabutylammonium fluoride (Alfa Aesar, Ward Hill, MA, USA), dibutyltin dilaurate (TCI, Tokyo, Japan), ammonium chloride (Samchun Chemical, Seoul, Korea), sodium sulfate (Samchun Chemical, Seoul, Korea), all solvents including dichloromethane, N-methyl-2-pyrrolidone, tetrahydrofuran, ethyl acetate, dimethyl sulfoxide, dimethylformamide (Samchun Chemical, Seoul, Korea) and deuterated solvents for nuclear magnetic resonance (Cambridge Isotope Laboratories, Tewksbury, MA, USA).

### 2.2. Methods

#### 2.2.1. Synthesis of Furan Carbonate Diols Protected with Tert-Butyldiphenylsilane (FCD-Ps)

The weighed protected ω-hydroxy bromoalkane (TBDPSO-R_1_-Br; P1~3) (P1 185 mg; P2 166 mg; P3 193 mg, 2.2 eq, 0.44 mmol) was transferred to an autoclave using a dichloromethane (DCM), and the DCM was removed using a vacuum. The BHMF (26 mg, 0.20 mmol), cesium carbonate (Cs_2_CO_3_; 261 mg, 0.80 mmol), and *N*-methyl-2-pyrrolidone (NMP; 0.50 mL) were consecutively added; the autoclave was then sealed and pressurized with CO_2_ to 10 bar. The resulting reaction mixture was stirred at 100 °C for 24 h. After the reaction, the autoclave was cooled to 25 °C and the CO_2_ was released. The product was purified using silica gel flash column chromatography (10% ethyl acetate in hexanes) to provide FCD-Ps as a colorless oil.

FCD-P1: R_1_ = hexylene. Yield 77 mg (43%). ^1^H NMR (500 MHz, DMSO-*d*_6_): δ (ppm): 7.60 (m, 8H), 7.43 (m, 12H), 6.54 (s, 2H), 5.08 (s, 4H), 4.05 (t, *J* = 6.35 Hz, 4H), 3.62 (t, *J* = 6.35 Hz, 4H), 1.56 (m, 8H), 1.34 (m, 8H) 0.98 (s, 18H). ^13^C NMR (125 MHz, DMSO-*d*_6_): δ (ppm): 154.70, 150.20, 135.47, 133.80, 130.23, 128.31, 112.63, 68.22, 63.73, 61.11, 32.25, 31.17, 28.49, 27.13, 25.27, 19.23.

FCD-P2: R_1_ = propylene. Yield 86 mg (53%). ^1^H NMR (500 MHz, DMSO-*d*_6_): δ (ppm): 7.59 (m, 8H), 7.42 (m, 12H), 6.56 (s, 2H), 5.09 (s, 4H), 4.24 (t, *J* = 6.15 Hz, 4H), 3.69 (t, *J* = 5.95 Hz, 4H), 1.85 (t, *J* = 6.05 Hz, 4H), 0.97 (s, 18H). ^13^C NMR (125 MHz, DMSO-*d*_6_): δ (ppm): 154.63, 150.16, 135.43, 133.44, 130.31, 128.34, 112.72, 65.13, 61.17, 60.19, 31.38, 27.04, 19.21.

FCD-P3: R_1_ = *p*-xylylene. Yield 14 mg (8%). ^1^H NMR (500 MHz, DMSO-*d*_6_): δ (ppm): 7.64 (m, 8H), 7.45 (m, 12H), 7.36 (s, 8H), 6.57 (s, 2H), 5.15 (s, 4H), 5.13 (s, 4H), 4.77, (s, 4H), 1.03 (s, 18H). ^13^C NMR (125 MHz, DMSO-*d*_6_): δ (ppm): 154.57, 150.13, 141.34, 135.46, 134.47, 133.29, 130.42, 128.79, 128.43, 126.36, 112.78, 69.53, 65.26, 61.38, 27.11, 19.33.

#### 2.2.2. Synthesis of the Furan Carbonate Diols (FCDs)

To a solution of FCD-P (0.074 mmol) in anhydrous THF (0.80 mL), tetrabutylammonium fluoride (TBAF; 1.0 M in THF, 0.16 mL, 0.16 mmol) was added at room temperature under N_2_. The mixture was stirred for 3 h at room temperature, quenched with a saturated aqueous ammonium chloride solution, and extracted with ethyl acetate (EtOAc × 3). The combined organic layers were dried over anhydrous sodium sulfate, filtered, and concentrated in vacuo. The residue was purified by flash silica gel column chromatography (3:1 hexanes/EtOAc) to afford FCD as a colorless oil.

FCD-1: R_1_ = hexylene. Yield 29 mg (95%). ^1^H NMR (500 MHz, DMSO-*d*_6_): δ (ppm): 6.55 (s, 2H), 5.09 (s, 4H), 4.34 (t, *J* = 5.05 Hz, 2H), 4.08 (m, 4H), 3.37 (m, 4H), 1.58 (m, 4H), 1.40 (m, 4H), 1.29 (m, 8H). ^13^C NMR (125 MHz, DMSO-*d*_6_): δ (ppm): 154.24, 149.72, 112.17, 67.83, 60.65, 32.34, 28.11, 25.08, 24.70. FTIR (cm^−1^): 3379 (O–H stretching vibration), 1745 (C=O stretching vibration), 1248 (C–O–C stretching vibration).

FCD-3: R_1_ = *p*-xylylene. Yield 5 mg (15%). ^1^H NMR (500 MHz, DMSO-*d*_6_): δ (ppm): 7.38 (m, 4H), 7.32 (m, 4H), 6.56 (s, 2H), 5.21 (t, *J* = 5.75 Hz, 2H), 5.13 (m, 10H), 4.50 (d, *J* = 5.5 Hz, 2H). ^13^C NMR (125 MHz, DMSO-*d*_6_): δ (ppm): 154.52, 150.11, 136.01, 128.76, 128.60, 126.94, 112.81, 69.28, 63.04, 61.42. FTIR (cm^−1^): 3330 (O–H stretching vibration), 1743 (C=O stretching vibration), 1236 (C–O–C stretching vibration).

#### 2.2.3. Synthesis of the Poly(Carbonate-co-Urethane) Networks (PCUs) via Ball-Milling

FCD-1 (416 mg, 1.0 mmol) or FCD-3 (456 mg, 1.0 mmol), erythritol (122 mg, 1.0 mmol), hexamethylene diisocyanate (HDI; 504 mg, 3.0 mmol) or 4,4′-diisocyanato-methylenedicyclohexane (HMDI; 787 mg, 3.0 mmol), and dibutyltin dilaurate (DBTDL; 1.0 mol%) were added in a 25 mL stainless steel vessel with two stainless steel balls (diameter, 15 mm each). The mixture was mounted on a mixer and vibrated at 20 Hz for 60 min. The product obtained was a yellow solid. FTIR (cm^−1^): 3344 (N–H stretching vibration), 2262 (N=C=O stretching vibration), 1725 (C=O stretching vibration), 1246 (NH–C=O stretching vibration).

#### 2.2.4. Fabrication of the PCU Films

The synthesized PCU was dispersed in dimethyl sulfoxide (DMSO) at a concentration of 10 wt%. The solution was transferred to a polyteterafluoroethylene (PTFE) Teflon mold (30 × 30 mm, 10 mm thickness), and the solvent was dried in an oven at 70 °C for 12 h. The dried sample was transferred to a stainless steel plate (12 × 12 mm, 0.8 mm thickness) and hot-pressed at 120 °C for 6 h. FTIR (cm^−1^): 3344 (N–H stretching vibration), 1725 (C=O stretching vibration), 1246 (NH–C=O stretching vibration).

### 2.3. Measurements

A 1A Retsch Mixer Mill MM 400 system (Haan, Germany) was used for the ball-milling experiments with a 25 mL stainless steel vessel and two stainless steel balls with a diameter of 15 mm each. The autoclave experiments employing a 50 mL stainless steel vessel were used as a system for applying pressure. Proton (^1^H) NMR spectra were recorded using a Bruker 500 MHz spectrometer (MA, USA). Carbon (^13^C) NMR spectra were recorded using a 125 MHz spectrometer with complete proton decoupling. The chemical shifts were reported in ppm (δ) with TMS as the internal standard; the coupling constants (*J*) were expressed in Hz. Column chromatography was carried out using a 100–200-mesh silica gel. Thin-layer chromatography analysis was performed on precoated silica gel 60 F_254_ slides (Merck, Darmstadt, Germany) and visualized by ultraviolet irradiation. Fourier transform infrared (FTIR) spectra were obtained using a spectrometer (Vertex70, Bruker Optics, MA, USA) equipped with a diamond attenuated total reflectance unit. The decomposition temperature (T_d_) of the polymer was determined by thermogravimetric analysis (TGA) using a Seiko Exstar 6000 (TG/DTA6100, Chiba, Japan). The measurements were carried out from 0 °C to 800 °C at a heating rate of 10 °C ∙min^−1^ under a flow of 100% nitrogen (N_2_). Differential scanning calorimetry (DSC) of the network powder was conducted using a DSC 7020 (Hitachi High-Tech, Tokyo, Japan) in temperatures ranging from −30 °C to 185 °C, 185 °C to −30 °C, and −30 °C to 185 °C with a heating/cooling rate of 5 °C∙min^−1^. The thermomechanical properties of films were characterized by dynamic mechanical analysis (DMA), which was carried out using a Seiko Exstar 6000 (DMA/SS6100, tension film mode). The temperature range was between −20 °C and 180 °C. The heating rate was 3 °C∙min^−1^ at a 1 Hz frequency. Rheological measurements of films were carried out using the 25-mm-diameter parallel plates of a modular compact rheometer (MCR 302e, Anton Paar, Graz, Austria). Stress–relaxation experiments were performed over a temperature range of 140 °C to 160 °C at a 3% strain, and the relaxation modulus was monitored as a function of time. A constant normal force of 5 N was applied to ensure good contact with the plate. Samples were held at the measurement temperature for 10 min at regular intervals. Tensile analyses were conducted at a tensile rate of 3 mm∙min^−1^ using a universal testing machine (UTM) (34SC-05, Instron, Norwood, MA, USA). The UTM samples were prepared on a stainless steel plate (5 × 50 mm, 0.8 mm thickness) in a hot press at 120 °C for 6 h. Thermomechanical Analysis (TMA) (TMA6100, Chiba, Japan) was measured at 3 °C∙min^−1^ from −35 °C to 200 °C for additional healing properties measurement. TMA samples (3 × 20 mm, 0.8 mm thickness) were prepared in the same way as UTM.

## 3. Results

### Synthesis of CO_2_-Immobilized Platform FCDs

**Scheme 1:**
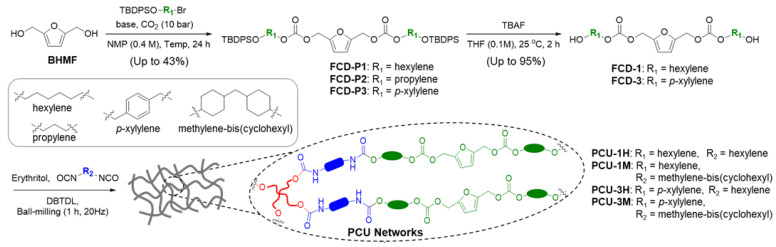
Synthesis of the PCU networks from CO_2_-immobilized, biomass-derived carbonate diols (FCDs).

The biomass-derived, CO_2_-immobilized platform FCDs were synthesized from biomass-derived diol BHMF via carbonation followed by a one-pot substitution reaction with a protected ω-hydroxy bromoalkane of three different moieties. The protected ω-hydroxy bromoalkanes (TBDPSO-R_1_-Br, P1~3) were prepared from an α,ω-dihydroxy alkane via bromination and silyl ether formation, or silyl ether protection on a ω-hydroxy-α-bromoalkane (Table S1). When CO_2_ was pressurized (10 bar) in an autoclave reactor containing BHMF and TBDPSO-R_1_-Br in the presence of a base, CO_2_ was successfully immobilized to BHMF with moderate yields (Scheme 1 and Table 1).

We set out to optimize the synthetic conditions for the direct introduction of CO_2_ using hexylene bromide P1 by screening bases using 8-diazabicyclo(5.4.0)undec-7-ene (DBU), potassium carbonate (K_2_CO_3_), and Cs_2_CO_3_ (Table 1 entries 1–3). The reaction did not proceed with DBU and the starting materials remained mostly (entry 1). Only 20% conversion was observed in the ^1^H-NMR analysis in the case of K_2_CO_3_ (entry 2). The reaction conversion with respect to BHMF was significantly higher (90%) when Cs_2_CO_3_ was used; however, the isolated yield was only 17% (entry 3). The isolated yield was marginally improved by increasing the equivalent bromide (entry 4) or lengthening the reaction time from 24 to 48 h (entry 4). For a higher yield, the reaction temperature was increased to 100 °C, resulting in a moderate yield of 43% at 24 h (entry 5). Other bromoalkanes (propylene P2 and xylylene P3) were also subjected to the optimized conditions, and the corresponding protected carbonate diols (FCD-Ps) were obtained in isolated yields of 53% and 8%, respectively (entries 6 and 7). The yield in the case of *p*-xylylene bromide P3 was unsatisfactory, and it appeared that benzylic bromides were not compatible with nucleophilic substitution by the carbonate intermediate, resulting from CO_2_ addition to the cesium alkoxide [54], or because unknown side reactions occurred.

The target FCDs were obtained through deprotection of FCD-Ps using TBAF (Scheme S1); however, only FCD-1 and FCD-3 were successfully isolated in yields up to 95%. In the case of FCD-2, which had a propylene group, it was confirmed by ^1^H-NMR that a cyclic reaction occurred during deprotection, and propylene carbonate was produced as a by-product. Although the overall yields were only moderate, it should be noted that an ecofriendly polymer platform was successfully prepared by directly introducing CO_2_ into a biomass-derived diol.

**Figure 2:**
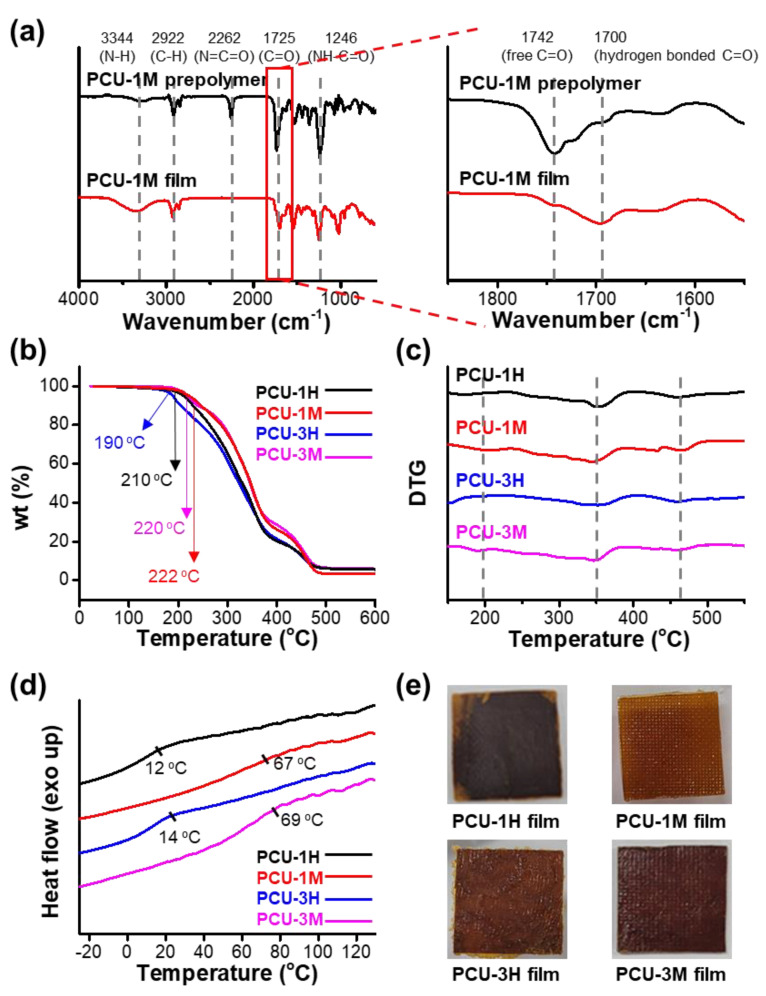
(**a**) Fourier transform infrared analysis of the PCU-1M prepolymer powder and the hot-pressed film, showing the complete conversion of the starting materials; (**b**) The TGA curves of films; (**c**) The DTG curves of the PCU films; (**d**) The DSC curves of the PCU films and the corresponding T_g_s; (**e**) Photographs of the PCU films after the hot-pressing process.

With FCDs at hand, the synthesis of biomass-derived PCU networks was attempted via a facile, ecofriendly mechanochemical method. Following the optimized conditions described elsewhere [52], a mixture including FCD, mesoerythritol, and diisocyanate (HDI or HMDI) was subjected to vibration ball-milling (20 Hz, 60 min) and subsequently hot-pressed (120 °C, 6 h, 30 MPa) to form urethane-bond networks (PCUs, Figure 1 and Table 2). The starting mixture comprised a ratio of diol:polyol(tetra-ol):diisocyanate = 1:1:3, which corresponded to the stoichiometric balance of [OH] and [NCO]. As shown in the PCU-1M spectra in the FTIR measurements (Figure 2a), immediately after the initial ball-milling, the portions of NCO moieties remained, suggesting that the reaction did not proceed completely, presumably because of an insufficient encountering of functional groups in the solid-state conditions. The NCO peak did not disappear, even when the ball-milling reaction was carried out for 2 h or more. These remainders became additional reaction sites during the hot-pressing. In fact, in the FTIR spectra, the stretching vibration of the NCO peak at 2262 cm^−1^ disappeared completely in the PCU-1M film, and the sharp peaks at 1725 and 1246 cm^−1^ were observed as emanating from the stretching vibrations of the urethane bond, C=O, and N–H, respectively. Additionally, the broad peaks at 3344 cm^−1^ were shown by N–H stretching vibrations. The stretching vibrations of C=O in the FTIR spectra of urethane polymers can form two peaks: the free C=O and the hydrogen bonded C=O. Since the hydrogen bonding to C=O may weaken the double bond character, the peak for C=O stretching can shift to a lower wavenumber than that of free C=O [55]. When the FTIR spectra of PCU-1M prepolymer and hot-pressed film in the range of wavenumbers 1600–1800 cm^−1^ were enlarged (Figure 2a), two peaks of C=O stretching were clearly presented. It can be assigned that the free C=O appears at 1742 cm^−1^ and the hydrogen bonded C=O appears at 1700 cm^−1^. Interestingly, the portions of the hydrogen bonded C=O appeared to increase in the hot-pressed film, which can be attributed to the fact that under hot pressing conditions, more urethane bonds are formed and polymer chains can be rearranged for more hydrogen bonding. Other PCU and PCU films also showed similar patterns to PCU-1M as a result of FTIR measurements (Figure S1).

We observed the swelling behavior of PCUs for up to 72 h in common laboratory solvents such as DMF, DMSO, and toluene (Figure S2). We observed that the swelling ratios of PCUs were very little in toluene (a few %), but relatively large in DMF and DMSO (up to 210%). Interestingly, hexylene-based PCU-1s showed the larger swelling ratios than p-xylylene-based PCU-3s; FCD moieties in PCUs seem to have more effect on swellings that diisocyanate parts. In addition, the dry weight after 72 h of swelling were measured, giving rise to a soluble fraction (ƒ_soluble_). In fact, we observed the ƒ_soluble_ of 2~41% of PCUs, which may suggest the portions of the incompletely crosslinked moieties. However, most of the masses (59~98%) appeared to remain after the swelling, which indicates that PCUs mostly have a good crosslinking network.

The film-production conditions were optimized through several trials to produce uniform films, thereby ensuring that all the starting materials were converted through the hot-press conditions (Figure 2e). As described above, the reaction did not proceed completely in the prepolymer state. The first step was to prepare a homogeneous state; the synthesized pre-PCU powder was dissolved in DMSO (10 wt%). The mixture was then transferred to a PTFE Teflon mold (30 × 30 mm) and dried in an oven (70 °C) for 12 h. The fabricated prefilm was placed in a stainless steel mold (30 × 5 mm, 0.8 mm thickness) and pressed for 6 h at 120 °C under a pressure of 30 MPa. Since pre-PCU powders synthesized with HMDI (PCU-Ms) were mixed homogeneously, the resulting films were also uniform during the hot press. However, pre-PCU powders made from HDI (PCU-Hs) did not dissolve or disperse well in the solution, and as a result, the film formation had to be performed in a rather heterogeneous state. Accordingly, slightly incongruent films were produced, presumably because the heat was not evenly distributed during the film formation.

Thermal properties of the CO_2_-immobilized, biomass-based PCU network polymers were tested through TGA and DSC (Figure 2b–d). For the TGA measurements, the samples were heated under an N_2_ atmosphere at 10 °C∙min^−1^, and the T_d5%_ was defined at the point where the 5% weight loss occurred. As shown in Figure 2b, the T_d5%_s were in the range of 190 °C–222 °C, and the pattern of weight loss appeared to be very similar for all the PCUs. We attributed the initial weight loss of 15%–20% at approximately 200 °C to the decomposition of the bis(oxymethyl)furan moiety, which had the lowest thermal stability [51]. Next, the second loss at nearly 230 °C means the carbamate and carbonate decompositions. The weight loss after 400 °C can be attributed to the thermal reduction of the diisocyanate parts (Figure 2c). Furthermore, the shapes of the TGA profiles were dependent on the structure of the diisocyanate moieties; HMDI-based PCU-Ms showed slightly higher thermal stability compared with HDI-based PCU-Hs.

The glass transition temperature (T_g_) of the PCU networks was measured during the first cooling range from 185 °C to −30 °C at a rate of 5 °C∙min^−1^ (Figure 2d). The HDI-based PCUs, such as PCU-1H and PCU-3H, showed lower T_g_s (12 °C and 14 °C, respectively) compared with the HDMI-based PCUs, e.g., PCU-1M and PCU-3M (67 °C and 69 °C). The bis(cyclohexyl) segments in HMDI appeared rather torsionally rigid and there was reduced rotational freedom in the main chain, thereby increasing the T_g_ [56]. However, despite torsional rigidity, PCU-Ms produced a more homogeneous state under hot-press conditions compared with PCU-Hs (see above), resulting in films that were mechanically more intact.

**Figure 3:**
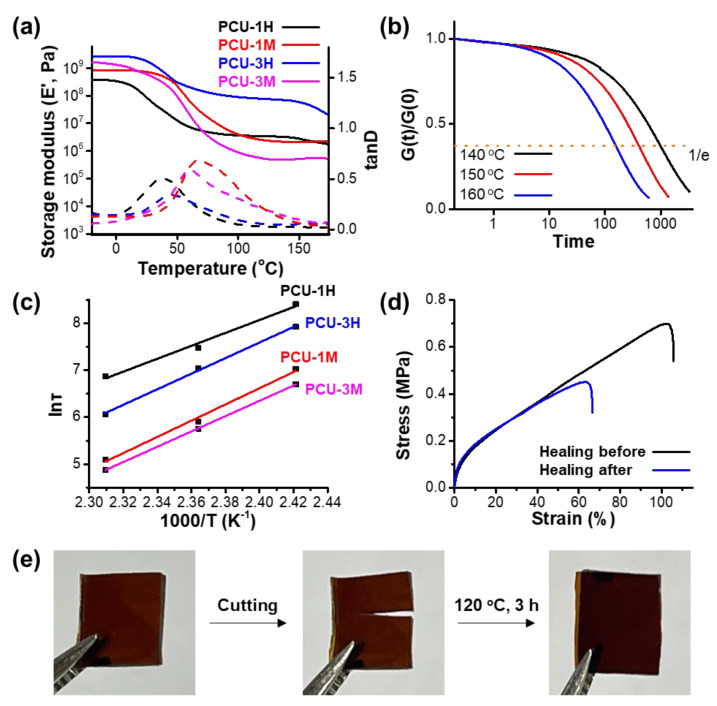
Thermomechanical properties of PCU films. (**a**) Tensile storage modulus and dissipation factor tan δ, measured using DMA from −20 °C to 180 °C under an N_2_ atmosphere at a heating rate of 3 °C∙min^−1^. (**b**) Normalized stress–relaxation curves as a function of time for three different isothermal temperatures from 140 °C to 160 °C. (**c**) Fitting of the relaxation times (τ) to the Arrhenius equation (see Supplementary Materials). (**d**) Tensile stress-strain curves before and after healing measured by UTM using the PCU-1M film. (**e**) Photographs showing thermal reparing of the cutted film of PCU-1M (120 °C, 3 h).

Thermomechanical properties were investigated using films from CO_2_-immobilized biomass-based PCU networks, which revealed their malleable and self-healing properties (Table 2 and Figure 3). The PCUs were fabricated into a 30 × 5 mm film (0.8 mm thickness) and characterized by DMA with the tension mode (heating rate 3 °C∙min^−1^, 1% strain, and 1 Hz vibration) (Figure 3a). As the temperature increased from −20 °C to 180 °C, the tensile storage modulus (E′) of PCU-1H and PCU-1M was initially maintained at 4.2 and 827 MPa, respectively, until it began decreasing at ~38 °C and 68 °C, respectively. Here, T_g_ was estimated to be the peak in tan δ (loss modulus E″/storage modulus E′). The difference in the T_g_ of PCU-1s was attributed to the difference in the isocyanate moieties; the PCU based on the torsionally rigid HMDI showed a higher T_g_, which was consistent with the trends indicated by the DSC measurements above. Similarly, this trend was observed in PCU-3H and PCU-3M (46 °C and 60 °C, respectively). Interestingly, the initial E′ seems to depend largely on the FCD components; the aromatic group-containing FCD-3 resulted in higher E′ values (2.5 and 1.5 GPa for PCU-3H and PCU-3M, respectively) compared with linear alkyl FCD-1 (0.38 and 0.83 GPa for PCU-1H and PCU-1M, respectively). Furthermore, HMDI-based PCU-Ms showed larger drops in E′ at above their T_g_ than HDI-based PCU-Hs (up to 2500 vs. 820 MPa). We suspected that in the flexible and elongated states above the T_g_, the interchain interactions arising from the linear alkyl groups in PCU-Hs tended to be stronger than those from the bis(cyclohexyl) groups in PCU-Ms.

After a decrease in the E′, the E′ of HDMI-based PCU films (PCU-1M and PCU-3M film) maintained a rubbery plateau at above ~140 °C. These plateaus suggested that the crosslinking density had been well-maintained and that the urethane (or carbonate) bond exchange may also have occurred as the temperature increased. Conversely, the E′ of HDI-based PCU films (PCU-1H and PCU-3H film) showed a tendency to decrease at 150 °C or higher; accordingly, it was inferred that the crosslinking density may have decreased. We suspect that the decrease in E′ of the PCU-Hs at extremely elevated temperatures had partly been due to the mechanically less-intact state of the HDI-based PCU films.

Stress–relaxation analyses were conducted to derive the dynamic covalent characteristics of the PCU networks. The sample sizes were 5 × 5 mm (0.8 mm thickness), and the shear storage modulus (G′) was monitored from 140 °C to 160 °C (10 °C intervals) while applying 3% strain and a constant normal force of 5 N as a function of time (Figure 3b). The normalized shear storage modulus [G(t)/G(0)] was plotted as a function of the relaxation time (t), and the representative stress–relaxation profiles for the rest of the PCU films are shown in Figure S3 and Table S2. According to the Maxwell relationship [G(t)/G0 = exp(−t/τ*)], the characteristic relaxation time (τ*) can be regarded as the time when the normalized modulus becomes a value of 1/e (≈0.37) [57,58,59]. When examining τ* at each temperature (T), these may exhibit Arrhenius behavior, a characteristic of typical associative CANs or vitrimers. By fitting ln τ* vs. 1000/T according to the Arrhenius equation, the kinetic activation energy (E_a_) can be obtained from the slopes of linear equation (Figure 3c). Using the relationship of viscosity (η) and τ*, topology freezing transition temperature (T_v_) was also calculated by the slope of the trend line. The T_v_ is a key transition temperature in CANs (refer to the calculation procedure of the Supplementary Materials). At the T_v_, the polymer network changes from a vitrified state to a deformable solid by a covalent-bond exchange reaction.

It seemed that the T_v_ of the PCU networks was largely determined by the diisocyanate moieties. The T_v_s of HDI-based PCU-Hs were much higher (104 °C–118 °C) than those of HMDI-based PCU-Ms (65 °C–72 °C). The difference in T_v_s appeared unclear because the same exchange reactions should have occurred in all PCUs; however, we suspect that the less-strong interchain interactions in PCU-Ms may have allowed for the faster exchange reactions. It should be noted that the dynamic bond exchange reactions in PCUs were clearly demonstrated by the rubbery plateaus in DMA and by the temperature-dependent stress–relaxation. Furthermore, it was shown that the thermomechanical properties of PCUs could be tuned via the choice of molecular structures in diol or diisocyanate moieties.

Based on the dynamic bond exchanges in PCUs, the self-healing properties of PCU-1M films were investigated through tensile testing using a UTM (Figure 3d,e). The PCU-1M film before healing was flexible and reflected a strain capacity of above 100% and tensile strength of 0.71 MPa. A portion of the PCU-1M film was cut and cured at 120 °C for 3 h to obtain a fully cured film (Figure 3e). This cured film showed a healing efficiency of approximately 60% (a recovered tensile strength of 0.43 MPa). In addition, performance measurement before and after healing was additionally measured using TMA, showing the original thermomechanical property was restored (Figure S4).

**Figure 4:**
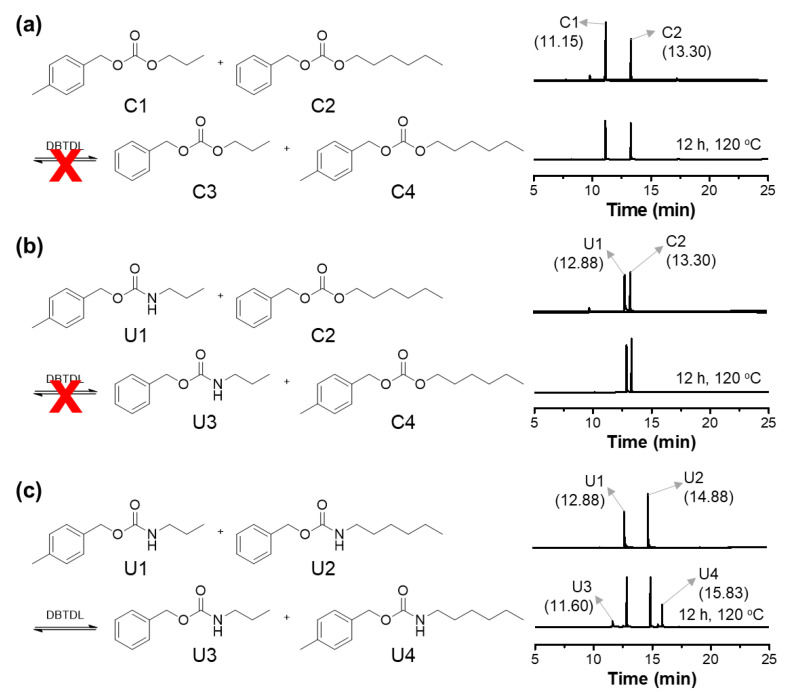
GC analyses during the exchange reactions (transcarbonation or transcarbamoylation) using the model compounds. (**a**) Exchange between carbonates (C1 + C2), resulting in no reaction. (**b**) Exchange between the carbonate and carbamate (U1 + C2), showing no change. (**c**) Exchange between carbamates (U1 + U2), and the appearance of new carbamates (U3 and U4).

Although a dynamic bond exchange reaction was demonstrated in PCUs, it is not clear whether transcarbamoylation (i.e., urethane exchange) or transcarbonation (i.e., carbonate exchange) occurred, or whether both reactions occurred simultaneously. To investigate which reaction occurred predominantly, we investigated the reactivity of the exchange reactions through a model-compound study using gas chromatography (GC) analysis (Figure 4). For the model exchange reactions, phenyl and toluene-functionalized carbamates (U1-4) and carbonates (C1-4) were synthesized with hexyl and propyl groups (each in 2 × 2 = 4 combinations). The exchange reactions were performed by combining model molecules in the presence of DBTDL at 120 °C. The reaction conditions were the same for the PCU synthesis, except that the reaction was performed in acetonitrile (ACN, ~33 mM). First, to check the exchange reactivity between carbonates, C1 and C2 were combined and the reaction proceeded for 12 h, resulting in no change in GC profiles (Figure 4a). When C1 and U2 were mixed and tested for the reactivity between carbonate and carbamate, the starting materials remained after the reaction and no product was found (Figure 4b). However, when carbamates U1 and U2 were combined, the reaction proceeded, and it was confirmed that the exchange products, U3 and U4, had been generated (Figure 4c). Therefore, evidence showed that the exchange reaction may occur only between carbamates (urethanes) in PCU networks, and could be responsible for the covalent adaptive characteristics.

## 4. Conclusions

In summary, a new CO_2_ utilization strategy was suggested, and FCDs were successfully synthesized by introducing CO_2_ directly into a biomass-derived diol BHMF. Using FCDs, PCU networks were prepared with erythritol and diisocyanate in a ratio of [OH]:[NCO] = 1:1 via facile ball-milling. The thermal and thermomechanical properties of PCUs were thoroughly investigated using TGA, DMA, and rheometry, and revealed the characteristics in the form of a CAN. It was shown that the molecular structures of FCDs (hexylene or *p*-xylylene) and diisocyantes (hexylene or (bis)cyclohexyl) affected the glass transition temperature, topology freezing temperature, and the rubbery storage modulus E′. In addition, the malleable, recyclable, and healable properties of the biomass-derived, CO_2_-immobilized PCUs were demonstrated, which arose due to the dynamic bond exchange reactions. It was confirmed via a model compound study that the bond exchanges occurred mainly between carbamates (not carbonates) under the specific reaction conditions. We believe our strategy presents an efficient approach for using biomass and CO_2_ simultaneously, and for producing sustainable polyurethanes and other copolymers.

## Figures and Tables

**Table 1:** Optimizing conditions for the synthesis of CO_2_-immobilized carbonate diol FCD-Ps ^1^.

Entry	R_1_	Base	Temp.	Yield ^2^
1	hexylene (2 eq)	DBU	80 °C	No reaction
2	hexylene (2 eq)	K_2_CO_3_	80 °C	20% ^3^
3	hexylene (2 eq)	Cs_2_CO_3_	80 °C	17%
4	hexylene (2.2 eq)	Cs_2_CO_3_	80 °C	25% (29% ^4^)
5	hexylene (2.2 eq)	Cs_2_CO_3_	100 °C	43%
6	propylene (2.2 eq)	Cs_2_CO_3_	100 °C	53%
7	*p*-xylylene (2.2 eq)	Cs_2_CO_3_	100 °C	8%

^1^ Reaction and condition: BHMF (0.2 mmol), base (4.0 eq), NMP (0.4 M) using an autoclave under 10 bar CO_2_. ^2^ Isolated yield. ^3^ NMR yield. ^4^ 48 h reaction.

**Table 2:** Physical properties of synthesized PCUs as different types of alcohol and isocyanate chains ^1^.

Entry	Alcohol (R_1_)	Isocyanate (R_2_)	T_g_^2^ (°C)	T_g_^3^ (°C)	T_d5%_^4^ (°C)	T_dmax_^5^ (°C)	tan δ	E′ ^6^ (MPa)	T_v_^7^ (°C)
PCU-1H	FCD-1	HDI (H)	38	12	210	490	0.35	4.2	104
PCU 1M	FCD-1	HMDI (M)	68	67	222	490	0.70	2.4	72
PCU-3H	FCD-3	HDI (H)	46	14	190	490	0.52	122	118
PCU-3M	FCD-3	HMDI (M)	60	69	220	490	0.60	0.54	65

^1^ Conditions: alcohol (FCD-1 or FCD-3, 1.0 eq), erythritol (1.0 eq), isocyanate (HDI or HMDI, 3.0 eq), DBTDL (1.0 mol%) using ball-milling 20 Hz for 60 min. ^2^ Defined as the temperature of the maximum in tan δ. ^3^ Determined by DSC for the first cooling range from 185 °C to −30 °C at a heating rate of 5 °C∙min^−1^. ^4^ Determined by the 5% weight-loss temperature from TGA. ^5^ Determined by the maximum temperature for fully degradation from TGA. ^6^ Determined by the rubbery plateau region in DMA curves. ^7^ Calculated using the Maxwell model and Arrhenius equation.

## Data Availability

The data presented in this study are available on request from the corresponding authors.

## Supplementary Materials

The following are available online at www.mdpi.com/article/10.3390/polym13244381/s1, T_v_ Calculations method Table S1: Synthesis of TBDPSO-R_1_-Br, Table S2: SRA of PCU-1H, PCU-3H and PCU-3M films, Scheme S1: Optimizing conditions for the deprotection of FCD-Ps, Figure S1: FTIR spectra of PCU-1H, PCU-3H, and PCU-3M in the prepolymer state and the film state, Figure S2: Swelling tests of PCU-1H, PCU-1M, PCU-3H, and PCU-3M, Figure S3: SRA of PCU-1H, PCU-3H, and PCU-3M through a rheometer, Figure S4: TMA of PCU-1M films before and after healing.
