# Supplementary material for: Self-Healable and Recyclable Biomass-Derived Polyurethane Networks through Carbon Dioxide Immobilization
Source: Polymers (Basel). 2021 Dec 14;13(24):4381. doi: 10.3390/polym13244381 (PMC8707029; doi:10.3390/polym13244381)
Supplement: Supplementary file 1 [file polymers-13-04381-s001.zip › polymers-1499239-supplementary.pdf]

Supplementary Materials

Self-Healable and Recyclable Biomass-Derived Polyurethane Networks through Carbon Dioxide Immobilization

Seohyun Baek [†], Juhyen Lee [†], Hyunwoo Kim, Inhwan Cha, and Changsik Song [*]

Department of Chemistry, Sungkyunkwan University, Suwon, Gyeonggi, 16419, Republic of Korea

*Email: songcs@skku.edu.

Table of Contents

1. Supplementary Figures and Tables

T_v calculation method		S2
Table S1.	Synthesis of TBDPSO-R_1-Br	S3
Scheme S1.	Optimizing conditions for the deprotection of FCD-Ps	S3
Figure S1.	FTIR spectra of PCU-1H, PCU-3H, and PCU-3M in the prepolymer state (a) and the film state (b)	S4
Figure S2.	Swelling tests of PCU-1H (a), PCU-1M (b), PCU-3H (c), and PCU-3M (d)	S5
Figure S3.	Stress relaxation analyses (SRA) of PCU-1H (a), PCU-3H (b), and PCU-3M (c) through a rheometer	S6
Table S2.	SRA of PCU-1H, PCU-3H and PCU-3M films	S6
Figure S4.	TMA of PCU-1M films before and after healing	S6

2. NMR spectra of the synthesized compounds S7

T_v Calculation Method

In order to obtain T_v, the relaxation time (τ) was plotted versus 1000/T according to the Arrhenius type equation (eq. 1) where R is the normal gas constant (8.314 $J{\cdot}K^{-1}mol^{-1}$), E_a is the activation energy, and T is the temperature. Specifics, the eq. 1 can be transformed to the linear function (eq. 2): y = ax + b. The E_a can be calculated from the slope in the plot of lnτ versus 1000/T.

$$\tau(T) = \tau_0 \cdot e^{\frac{E_a}{R \cdot T}} \qquad \text{(eq. 1)}$$

$$ln\tau(T) = \frac{E_a}{R \cdot T} + ln\tau_0 = a \cdot \frac{1000}{T} + b \qquad \text{(eq. 2)}$$

The T_v is the parameter of temperature for vitrimer materials, below which the bond exchange reaction does not occur and they maintain their topology like a frozen state. This means that when the material undergoes the "liquid"-to-"solid" transition, the viscosity (η) reaches 10^{12} Pa. The equation between the viscosity (η) and the stress relaxation time (τ) follows the Maxwell relation (eq. 3), when the E' is the equilibrium storage modulus at rubbery plateau. Using (eq. 2) and (eq. 3), T_v can be derived from (eq. 4).

$$\eta = \frac{1}{3} \times E' \times \tau \qquad \text{(eq. 3)}$$

$$T_v = \frac{1000 \cdot a}{\ln\left(\frac{3 \cdot \eta}{E'}\right) - b} \qquad \text{(eq. 4)}$$

Table S1. Synthesis of TBDPSO-R_1-Br[a]

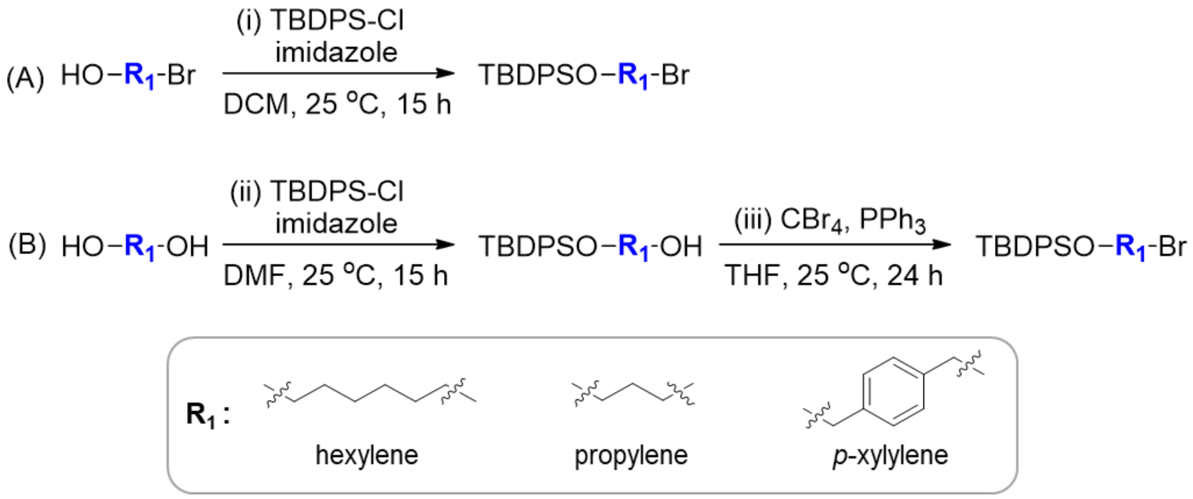

Entry	Method	R_1	Yield[b]
1	A	hexylene	89%
2	A	propylene	87%
3	B	*p*-xylylene	99%[C]

[a]Condition: (Method A) (i) HO-R-Br (1.0 eq), TBDPS-Cl (1.1 eq), imidazole (1.5 eq), DCM (0.70 M), under an N_2 atmosphere. (Method B) (ii) HO-R-OH (4.0 eq), TBDPS-Cl (1.0 eq), imidazole (3.1 eq), DMF (0.10 M), under an N_2 atmosphere, (iii) TBDPSO-R_1-OH (1.0 eq), CBr_4 (1.5 eq), PPh_3 (1.5 eq), THF (0.10 M), under an N_2 atmosphere. [b]Isolated yield. [C]overall isolated yield.

Scheme S1. Optimizing conditions for the deprotection of FCD-Ps.

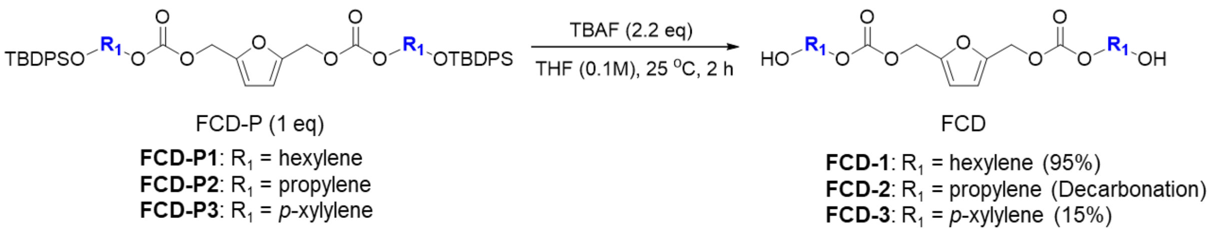

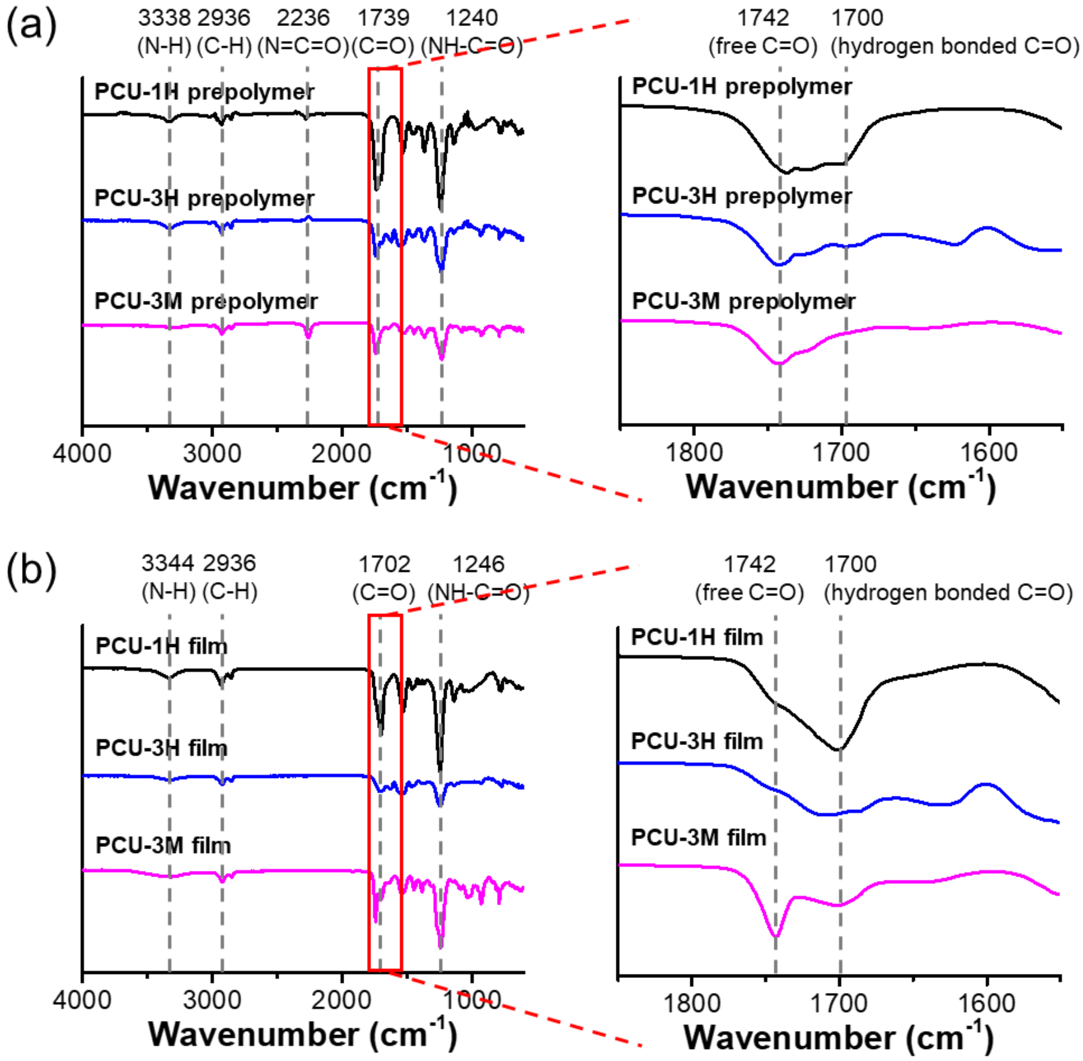

Figure S1. FTIR spectra of PCU-1H, PCU-3H, and PCU-3M in the prepolymer state (a) and the film state (b)

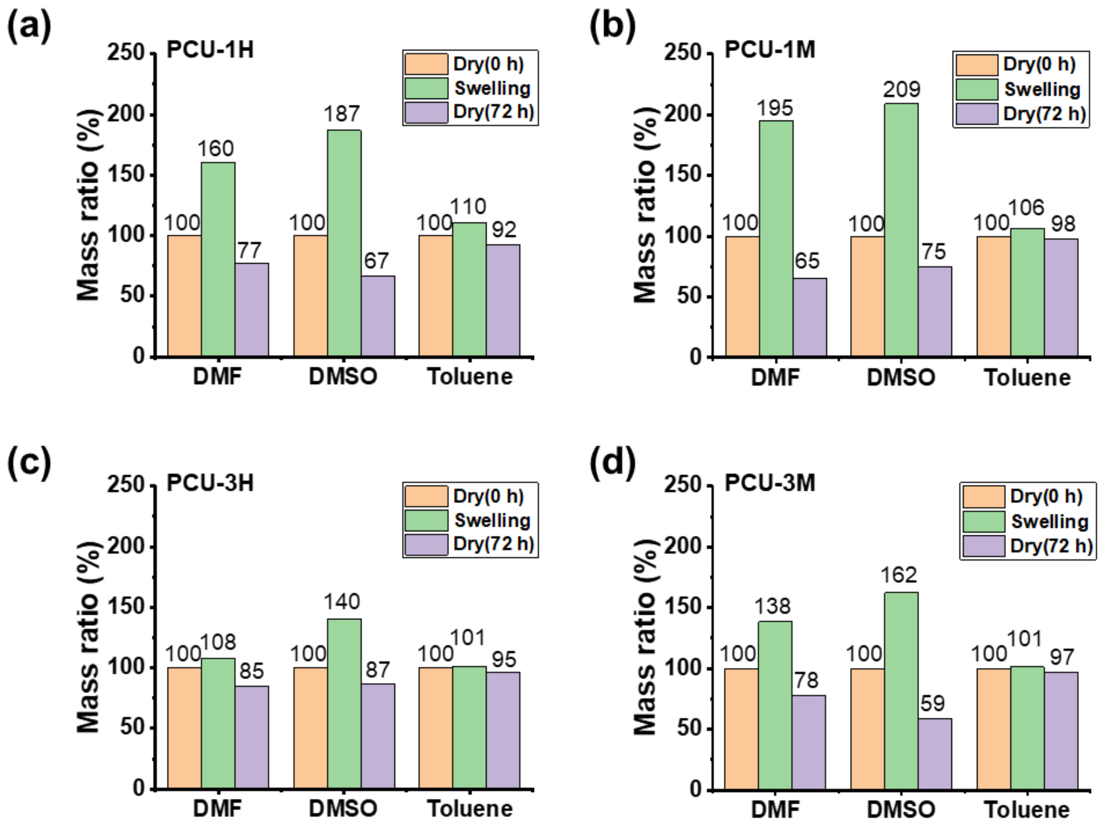

(e)

	DMF		DMSO		Toluene	
	$d_{swelling}$	$f_{soluble}$	$d_{swelling}$	$f_{soluble}$	$d_{swelling}$	$f_{soluble}$
PCU-1H	60	23	87	33	10	8
PCU-1M	95	35	109	25	6	2
PCU-3H	8	15	40	13	1	5
CPU-3M	38	22	62	41	1	3

$$d_{swelling} = \frac{m_{swelling} - m_{dry(0h)}}{m_{dry(0h)}} \times 100\% \quad f_{soluble} = \frac{m_{dry(0h)} - m_{dry(72h)}}{m_{dry(0h)}} \times 100\%$$

Figure S2. Swelling tests in common laborary solvents (DMF, DMSO, and toluene) by measuring the masses of the dry state at 0 h (orange) and at 72 h (grey), as well as the solvent-uptaken state (green) of (a) PCU-1H, (b) PCU-1M, (c) PCU-3H and (d) PCU-3M at room temperature. The swelling degree ($d_{swelling}$) and the solublity fractions ($f_{soluble}$) were presented in the table (e).

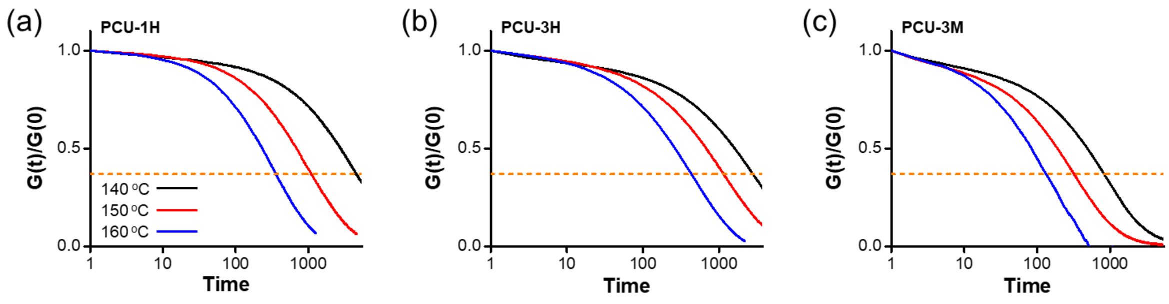

Figure S3. SRA of PCU-1H (a), PCU-3H (b), and PCU-3M (c) through a rheometer

Table S2. SRA of PCU-1H, PCU-3H and PCU-3M films

Entry	Storage modulus (E′)	a	b
PCU-1H	4.2 MPa	22.42	-45.94
PCU-3H	122 MPa	16.62	-32.29
PCU-3M	0.54 MPa	16.33	-32.85

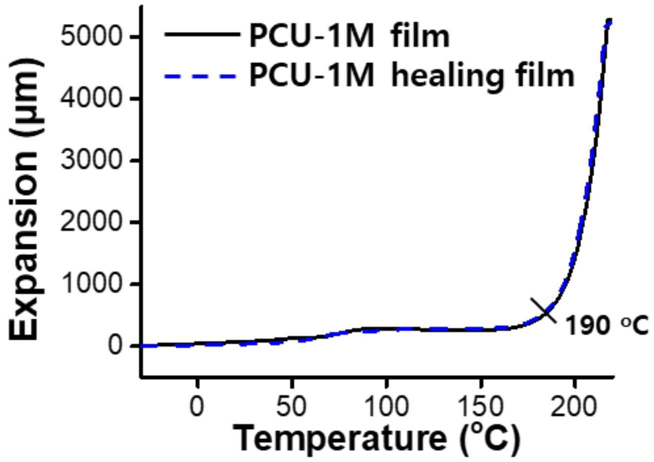

Figure S4. TMA of PCU-1M film before and after healing

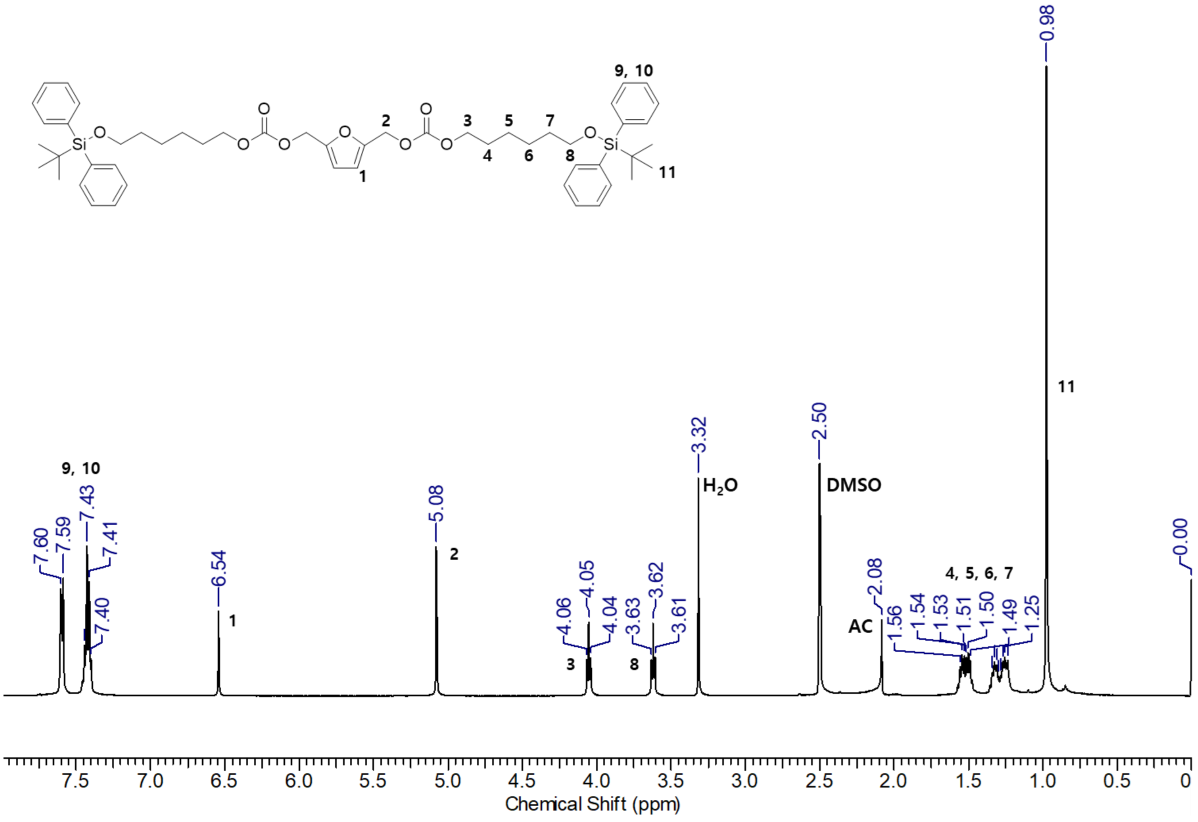

Figure S5. ^{1}H NMR spectrum of **FCD-P1** (500 MHz, DMSO-d_6)

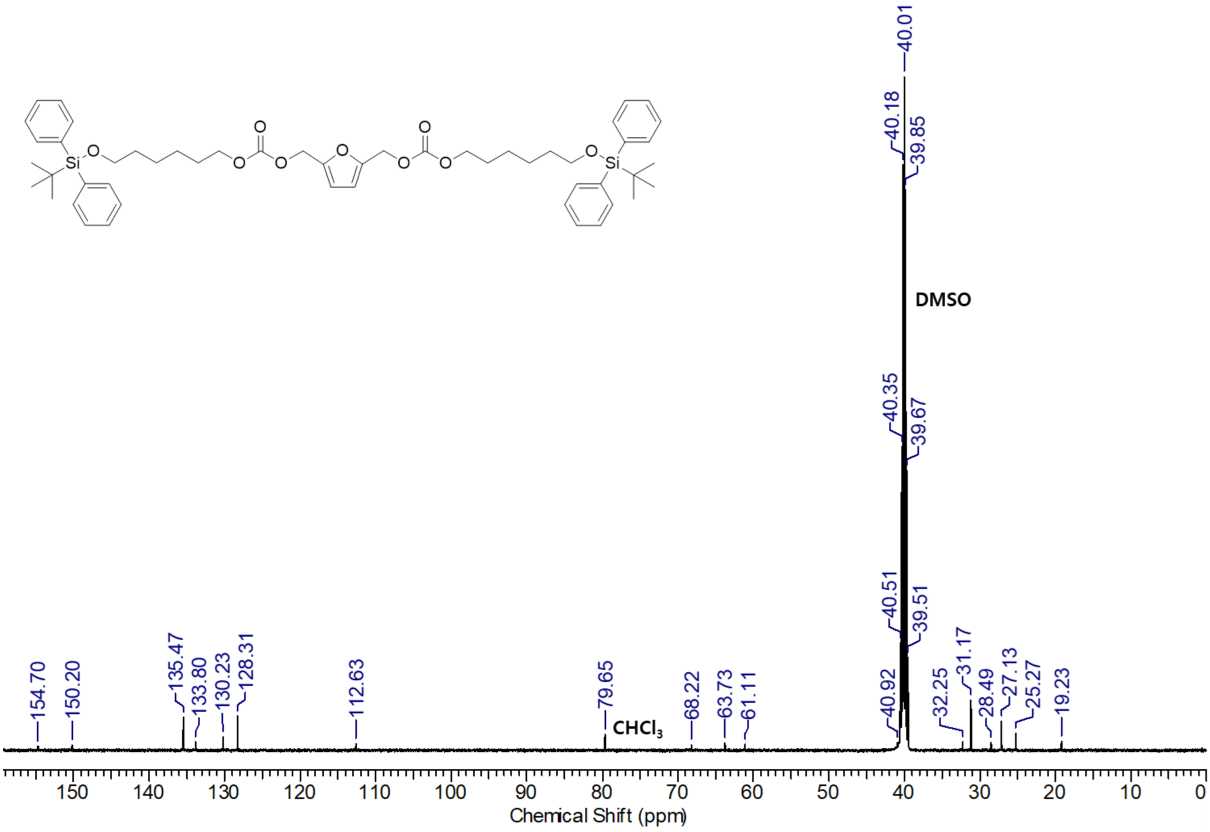

Figure S6. ^{13}C NMR spectrum of **FCD-P1** (125 MHz, DMSO-d_6)

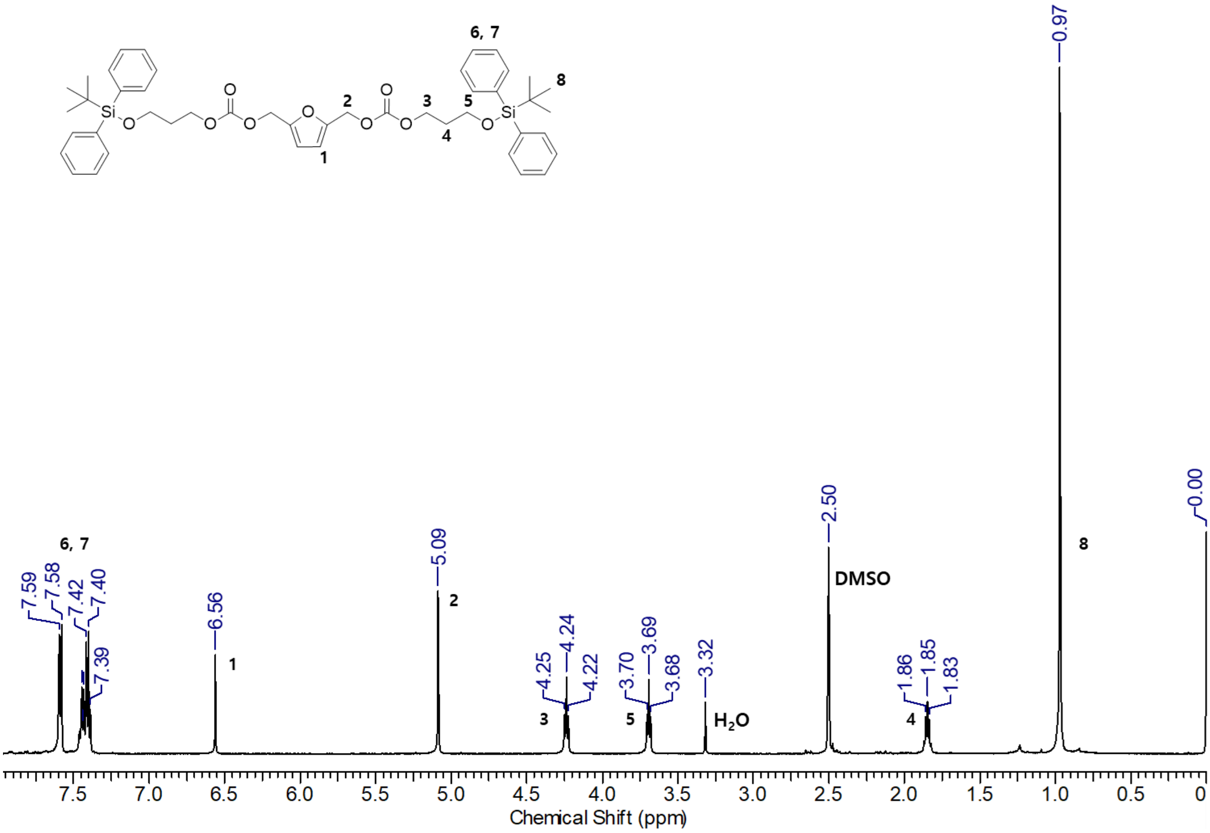

Figure S7. ^{1}H NMR spectrum of **FCD-P2** (500 MHz, DMSO-d_6)

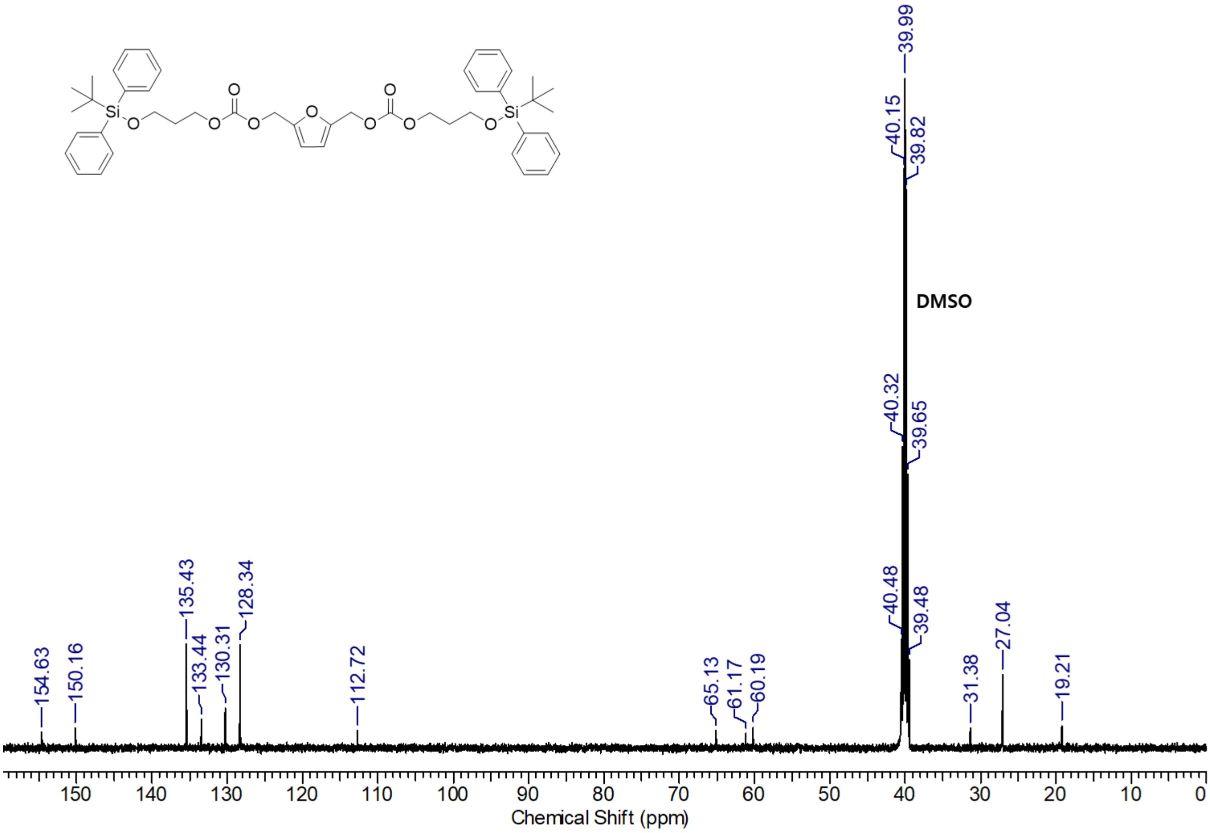

Figure S8. ^{13}C NMR spectrum of **FCD-P2** (125 MHz, DMSO-d_6)

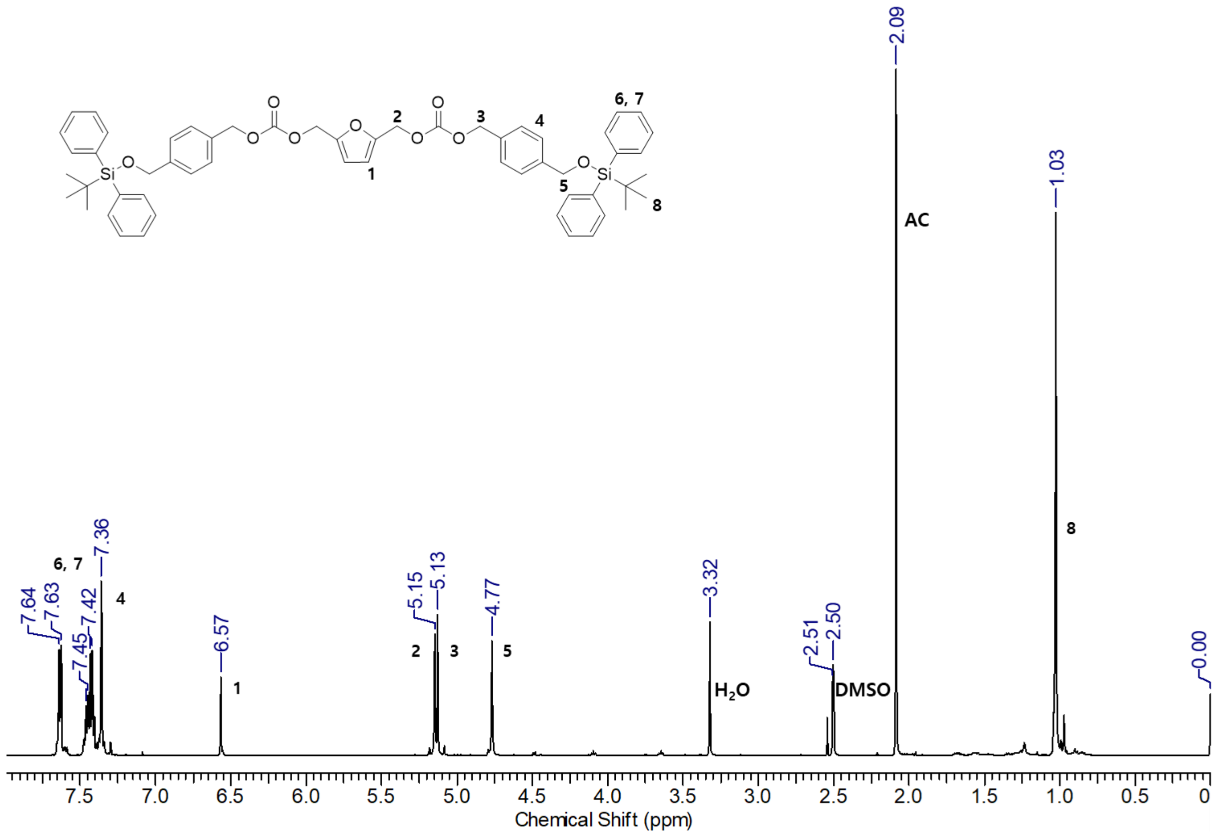

Figure S9. ^{1}H NMR spectrum of **FCD-P3** (500 MHz, DMSO-d_6)

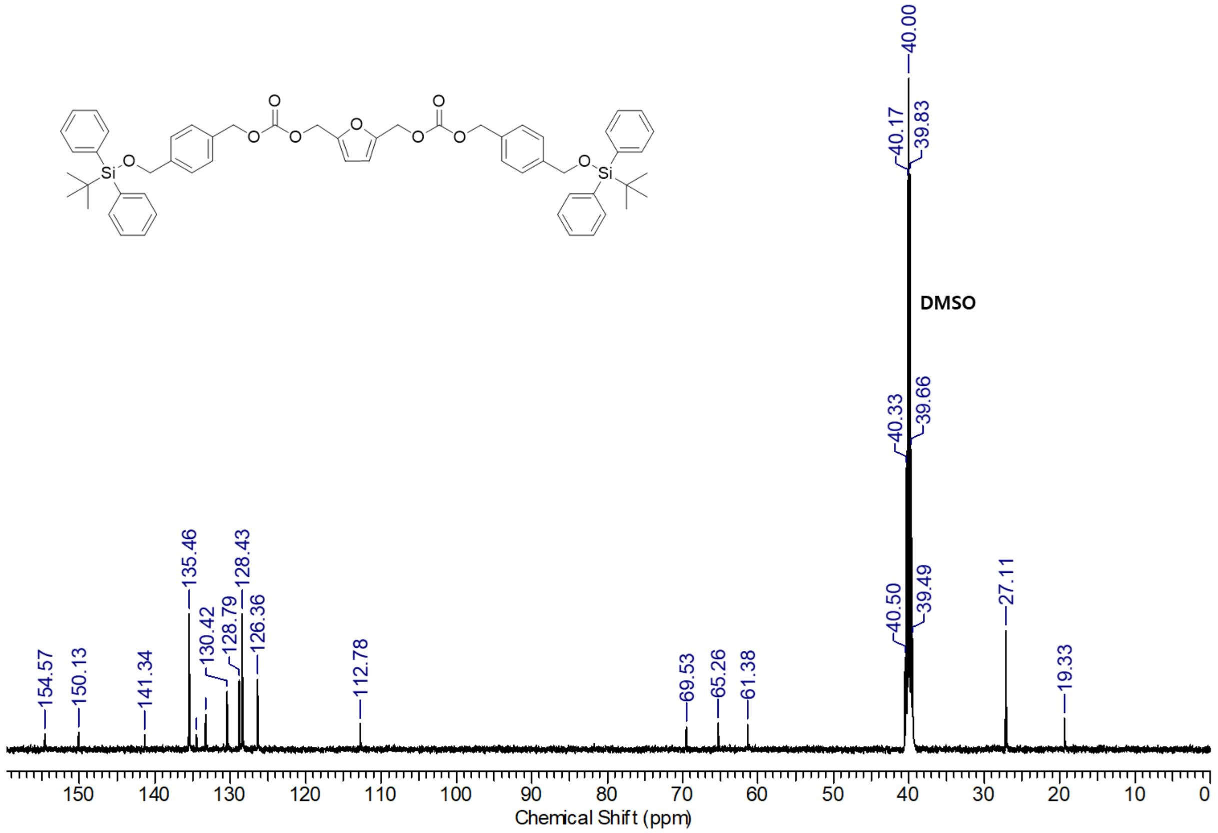

Figure S10. ^{13}C NMR spectrum of **FCD-P3** (125 MHz, DMSO-d_6)

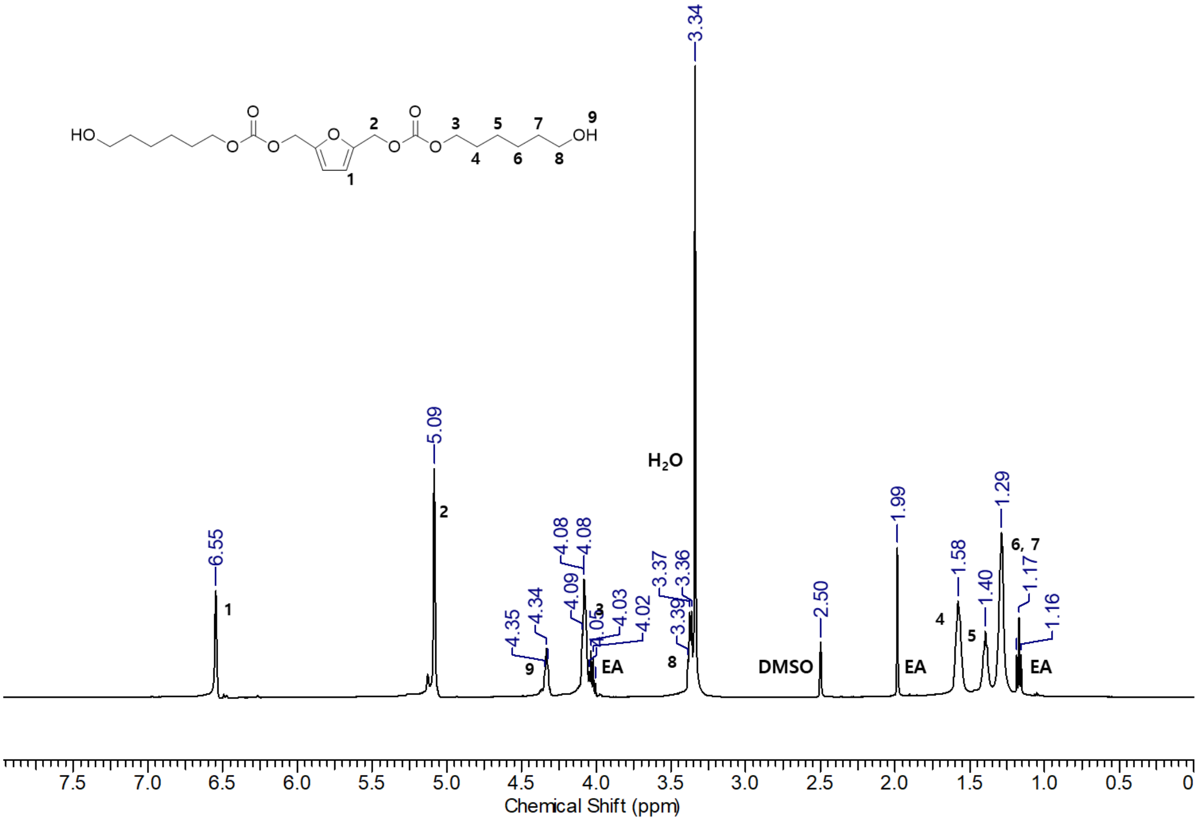

Figure S11. ^{1}H NMR spectrum of **FCD-1** (500 MHz, DMSO-d_6)

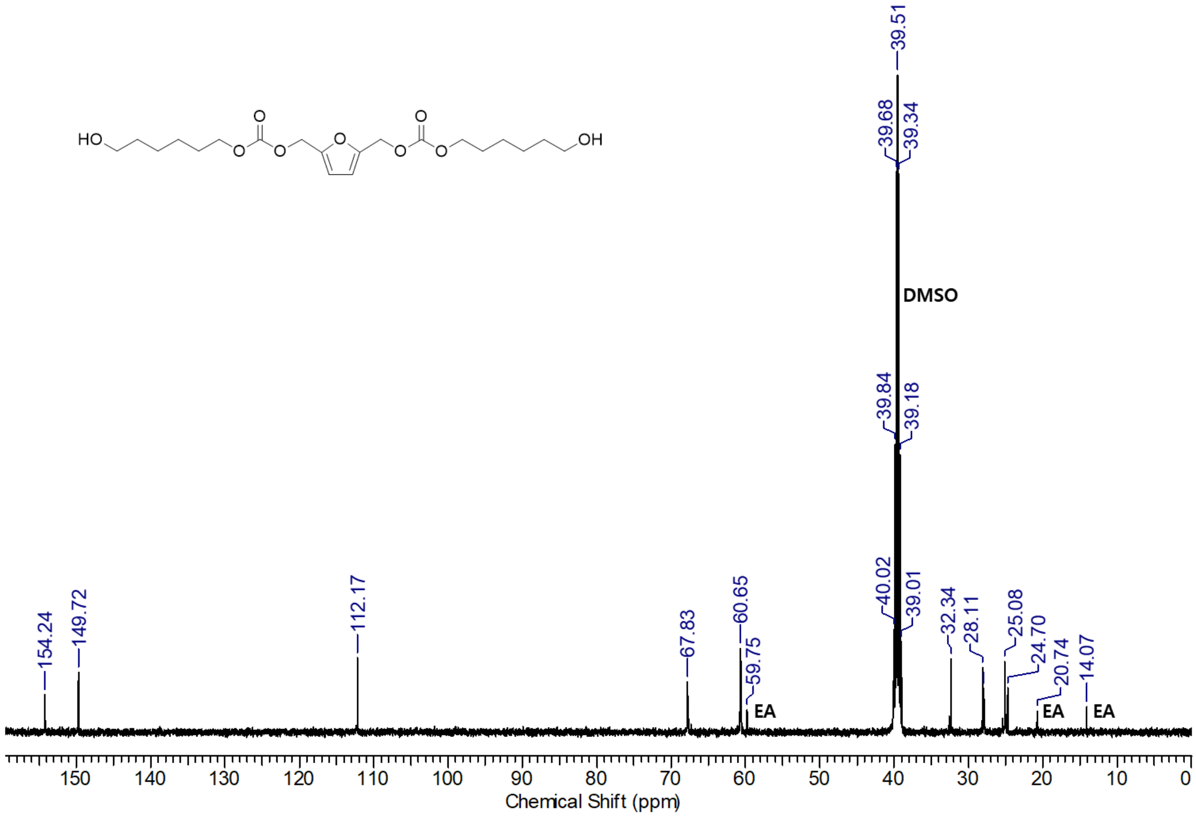

Figure S12. ^{13}C NMR spectrum of **FCD-1** (125 MHz, DMSO-d_6)

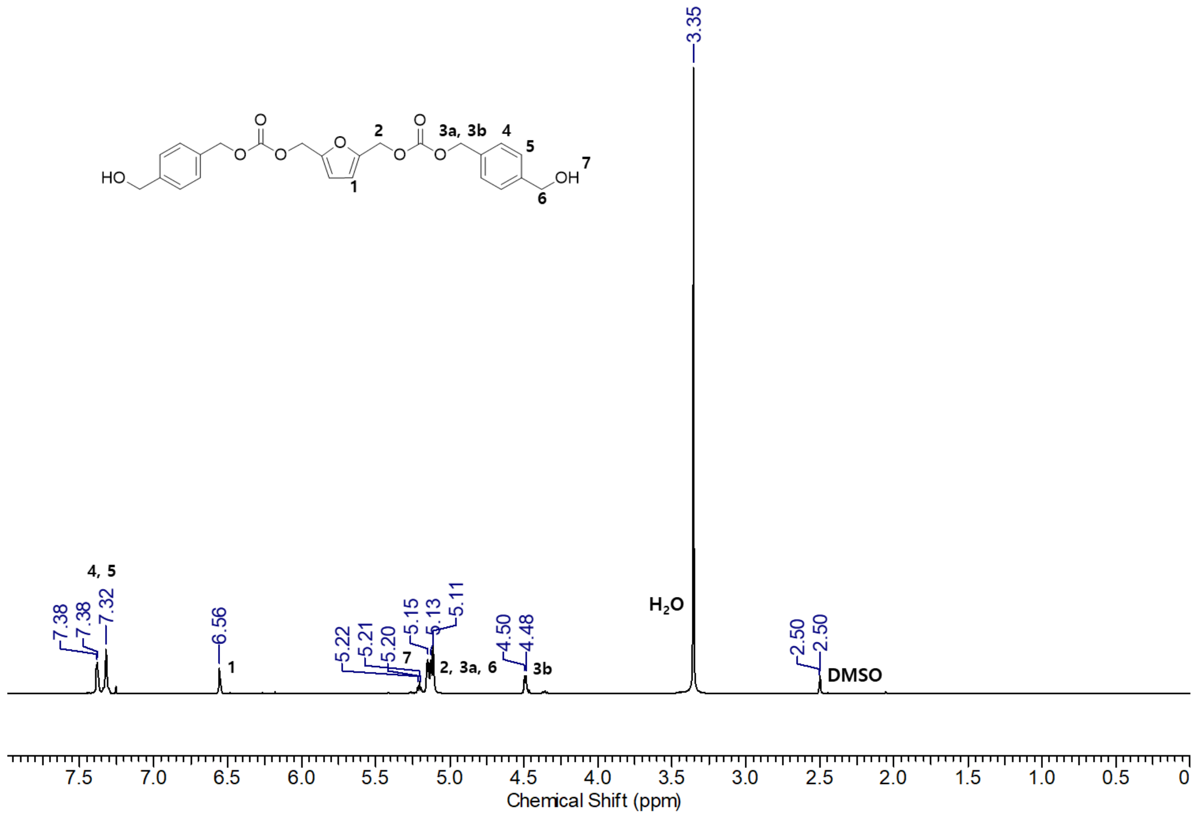

Figure S13. ^{1}H NMR spectrum of **FCD-3** (500 MHz, DMSO-d_6)

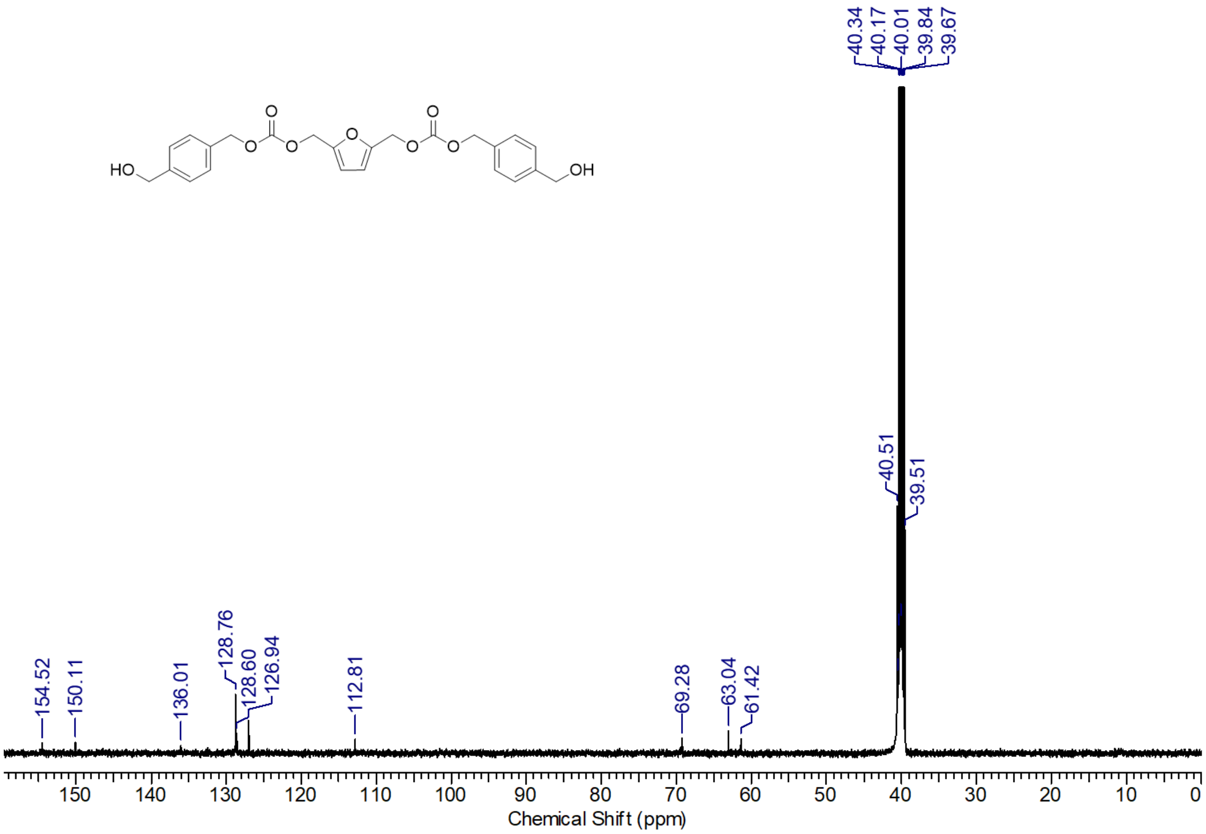

Figure S14. ^{13}C NMR spectrum of **FCD-3** (125 MHz, DMSO-d_6)
